# Cardioprotective Effects of Nanoemulsions Loaded with Anti-Inflammatory Nutraceuticals against Doxorubicin-Induced Cardiotoxicity

**DOI:** 10.3390/nu10091304

**Published:** 2018-09-14

**Authors:** Vincenzo Quagliariello, Raffaele Vecchione, Carmela Coppola, Chiara Di Cicco, Alberta De Capua, Giovanna Piscopo, Rolando Paciello, Viviana Narciso, Carmen Formisano, Orazio Taglialatela-Scafati, Rosario Vincenzo Iaffaioli, Gerardo Botti, Paolo Antonio Netti, Nicola Maurea

**Affiliations:** 1 Division of Cardiology, Istituto Nazionale Tumori–IRCCS-Fondazione G.Pascale, 80131 Napoli, Italy; melinacoppola@libero.it (C.C.); gpiscopo84@gmail.com (G.P.); rolando.paciello@unina.it (R.P.); n.maurea@istitutotumori.na.it (N.M.); 2 Center for Advanced Biomaterial for Health Care (CABHC), Istituto Italiano di Tecnologia, Largo Barsanti e Matteucci 53, 80125 Naples, Italy; chiara.dicicco@iit.it (C.D.C.); alberta.decapua@iit.it (A.D.C.); Paolo.Netti@iit.it (P.A.N.); 3 Department of Pharmacy, University of Naples Federico II, via D. Montesano 49, 80131 Naples, Italy; viviana.narciso@unina.it (V.N.); carmen.formisano2@unina.it (C.F.); scatagli@unina.it (O.T.-S.); 4 Department of Medical Oncology, Istituto Nazionale Tumori–IRCCS-Fondazione G.Pascale, 80131 Napoli, Italy; rv.iaffaioli@gmail.com; 5 Scientific Direction, Istituto Nazionale Tumori–IRCCS-Fondazione G.Pascale, 80131 Napoli, Italy; g.botti@istitutotumori.na.it

**Keywords:** cardiology, nanomedicine, nutraceuticals, doxorubicin, cytokines

## Abstract

Doxorubicin is a highly active antineoplastic agent, but its clinical use is limited because of its cardiotoxicity. Although nutraceuticals endowed with anti-inflammatory properties exert cardioprotective activity, their bioavailability and stability are inconsistent. In an attempt to address this issue, we evaluated whether bioavailable nanoemulsions loaded with nutraceuticals (curcumin and fresh and dry tomato extracts rich in lycopene) protect cardiomyoblasts (H9C2 cells) from doxorubicin-induced toxicity. Nanoemulsions were produced with a high-pressure homogenizer. H9C2 cells were incubated with nanoemulsions loaded with different nutraceuticals alone or in combination with doxorubicin. Cell viability was evaluated with a modified MTT method. The levels of the lipid peroxidation products malondialdehyde (MDA) and 4-hydroxy-2-butanone (4-HNA), and of the cardiotoxic-related interleukins IL-6, IL-8, IL-1β and IL-10, tumor necrosis factor-alpha (TNF-α), and nitric oxide were analyzed in cardiomyoblasts. The hydrodynamic size of nanoemulsions was around 100 nm. Cell viability enhancement was 35–40% higher in cardiomyoblasts treated with nanoemulsion + doxorubicin than in cardiomyoblasts treated with doxorubicin alone. Nanoemulsions also protected against oxidative stress as witnessed by a reduction of MDA and 4-HNA. Notably, nanoemulsions inhibited the release of IL-6, IL-8, IL-1β, TNF-α and nitric oxide by around 35–40% and increased IL-10 production by 25–27% versus cells not treated with emulsions. Of the nutraceuticals evaluated, lycopene-rich nanoemulsions had the best cardioprotective profile. In conclusion, nanoemulsions loaded with the nutraceuticals described herein protect against cardiotoxicity, by reducing inflammation and lipid oxidative stress. These results set the stage for studies in preclinical models.

## 1. Introduction

Doxorubicin is a cytotoxic antibiotic that is used to treat leukemia and lymphoma, as well as breast, lung, and other solid tumors. However, its clinical use is limited because of its irreversible cardiotoxicity that can lead to heart failure in a dose-dependent manner [1,2,3]. The mechanism of doxorubicin-induced cardiotoxicity is based on the generation of free radicals and reactive oxygen species that attach to membrane lipids and proteins thereby generating toxic products [4,5]. Doxorubicin stimulates overproduction of reactive oxygen species and impairs cardiac cell function [2]. It induces cardiotoxicity also by producing the pro-inflammatory interleukins IL-8 and IL-6 [6]. Natural products endowed with antioxidant activity, namely, survivin, sesamol and herbaleonurin, decrease or prevent doxorubicin-induced damage [7]. The natural antioxidants vitamin E and catalase, and the synthetic antioxidants 3-methyl-1-phenyl-2-pyrazolin-5-one (edaravone) are used to treat cardiac hypertrophy. The mechanism of action of nutraceuticals is based on the reduction of oxidative stress, cardiovascular risk factors such as Metabolic Syndrome [8,9] but their therapeutic potential in cardiology remains unknown [10,11].

Lycopene is a potent dietary carotenoid antioxidant thanks to its many conjugated double bonds [12] and it has the strongest singlet oxygen-quenching ability of all dietary carotenoids [13]. The relationship between lycopene and cardiovascular disease has been examined in several epidemiological studies [5]. The few studies conducted so far regarding the effect of lycopene or tomato extract on doxorubicin-induced cardiotoxicity showed some protective effects, but more studies are needed to evaluate their potential as cardioprotectors under doxorubicin treatment [14]. Curcumin is the main active ingredient of the turmeric spice *Curcuma longa*; it has several pharmacological and biological properties being an antioxidant, anticancer, anti-inflammatory and antiviral agent [12]. Curcumin induces apoptosis, via deactivation of NF-kB and its regulated gene products, and it also suppresses inflammatory cytokines such as interleukins (IL-1, -1β, -6, and -8), TNF-α, and COX-2 [15]. In studies comparing free and nano encapsulated curcumin, the bioavailability and effect of curcumin were greatly increased in terms of anti-inflammatory action [16,17]. The mechanism of action of these nutraceuticals are based on cell cycle regulation, tyrosine kinase modulation [18] inhibition of cytokine and interleukin secretion, and AMPK-Sclerosis Tuberous Complex (TSC) activation with consequent AKT-mTOR axis inhibition [19]. However, the clinical use of these nutraceuticals is limited by their low oral bioavailability due to oxidation in biological environments, which results in a low accumulation in the cell cytoplasm [20]. Given these limitations, the aim of this study was to encapsulate such nutraceuticals in nanocarriers able to protect them from oxidative and enzymatic environmentsin order to achieve a more specific and controlled release. We also investigated the pathways involved in cardioprotection by analyzing the anti-inflammatory and anti-oxidant effects of the nanocarriers based onIL-1β, IL-8, IL-6 and malondialdehyde (MDA)/4-HNA cellular secretion under pro-inflammatory conditions.

The proposed strategy of cardioprotection could be of translational importance in cardioncology considering the crucial role of the heart and vascular microenvironments in the etiology of cardiotoxicity.

## 2. Materials and Methods

### 2.1. Synthesis and Characterization of Nanoemulsions

Materials: Both soybean oil (density at 20 °C of 0.922 g mL^−1^) and surfactant Lipoid E80 (egg lecithin powder 80–85% enriched with phosphatidyl choline (PC) and 7–9.5% content in phosphatidyl ethanolamine (PE)) were purchased from Lipoid GmbH and used without further purification. Millipore Milli-Q water was used for the preparation of all nanoemulsions and solutions. Chitosan (CT, LMW 90–150 kDa, DDA 75–85%) was purchased from Sigma Aldrich (Milan, Italy). Chitosan was soluble in acidic conditions due to the protonation of its amine moieties [21] and was used without further purification; Fluorescein isothiocyanate (FITC, M.W. = 389.38 g mol^−1^) was also purchased from Sigma Aldrich and used without further purification. Curcumin (from *Curcuma longa* Turmeric, powder, M.W. = 368.38 g mol^−1^) was again purchased from Sigma-Aldrich and used with no further purification.

### 2.2. Preparation of Nanoemulsions

Nanoemulsions were prepared following a previously developed protocol [22] which can also be adapted to the encapsulation of contrasting agents [23]. Briefly, first the oil phase was prepared by adding the surfactant to the soybean oil and mixed at 60 °C under gentle stirring. Then, the oil phase was added drop wise to the water phase (Milli-Q water) and mixed using the immersion sonicator (Ultrasonic Processor VCX500 Sonic and Materials) until there was a suitable dissolution. The pre-emulsions were finally passed at 2000 bar through the high-pressure valve homogenizer (Microfluidics M110PS) for the first three individual cycles to greatly reduce the initial size, then the reservoir was continuously refilled for 200 steps. This method was used for the preparation of all oil-in-water nanoemulsions at 20 wt% of oil concentration. In particular, 5.8 g of surfactant were dissolved in 24 mL of oil. In the case of curcumin, 100 mg of drug were added to 24 mL of the oil phase dissolving the surfactant, and mixing at 60 °C under gentle stirring. Instead, in the case of tomato peel extracts, 5.8 g of surfactant were dissolved in 24 mL of oil already containing the biomolecule always mixing at 60 °C under gentle stirring. In fact, in the case of tomato peel extracts, in one option, ~800 g of fresh tomato peels were processed overnight with ~1.5 L of soy-bean oil in the Naviglio extractor which is a type of solid-liquid extractor [24]. This method has already been used to extract pure lycopene by using an alcoholic extraction [25]. In this case, for the first time we extracted the bioactive compounds from the tomato peels by using a vegetable oil as solvent that is then used for the preparation of the nanoemulsion. In a second option, the same amount of fresh tomato peels was first lyophilized and then processed by using the same procedure as for the fresh tomato peels. Fluorescent nanoemulsions were obtained by mixing 4 mL of ethanol solution of FITC (0.75 mg/mL) to the soybean oil during the emulsion preparation and drying the ethanol from the mixture with mild heating. The final concentration of FITC in the 10 wt% emulsion is 125 μg/mL. Then, 0.1 M acetic acid solution of chitosan (0.125 wt%) was prepared. Each nanoemulsion (freshTom-Ne, dryTom-Ne, curc-Ne and FITC-Ne) 20 wt% oil was added to the chitosan solution quickly under vigorous stirring and kept under stirring for 15 min to allow uniform chitosan deposition. Final concentrations of oil and chitosan were 10 and 0.1 wt%, respectively, while the pH of the final secondary nanoemulsions was 4. These nanoemulsions were re-dispersed using the method reported previously [26] and stored at room temperature. The nutraceutical-loaded nanoemulsions tested in this study are listed in Table 1.

### 2.3. LC/MS Analysis for Lycopene Measurement

HPLC-grade acetonitrile, methanol, dichloromethane, n-hexane and lycopene analytical standard were purchased from Sigma (St. Louis, MO, USA). The assay was performed according to the procedure described by Kozukue and Friedman (2003) slightly modified [27]. HPLC analysis was performed on a Jasco Extrema LC-4000 system (Jasco Inc., Easton, MD, USA) equipped with photo diode-array detector and autosampler. Data were acquired and analyses were performed using JASCO ChromNAV (version 2.02.04). Samples were analyzed on a Gemini C18 column (250 × 4.6 mm, 5 µm, Phenomenex) and lycopene was detected at 450 nm. The column was eluted at a flow rate of 1 mL/min with a two-solvent system, namely, (A) acetonitrile, (B) n-hexane/dichloromethane/methanol (1:1:1) with 82–76% A for the first 10 min, then in 2 min 58% A, in 6 min 40% A, and finally returned to 82% A in 5 min. This was followed by isocratic elution for 2 min. Calibration curve was performed with lycopene samples prepared freshly on a daily basis. The retention time of lycopene was 15 min. Each analysis was performed in triplicate. Lycopene standard was diluted to obtain the following ppm concentrations: 1, 5, 25, 50 and 100. A good linear fit range was found, the regression equation and correlation coefficient were respectively: y = 11660x + 776.75, R = 0.9998.

### 2.4. Cell Viability

To evaluate the cardioprotective effects of nutraceutical-loaded nanoemulsions on H9c2 cardiomyoblasts (American Type Culture Collection, Manassas, VA, USA), we measured the mitochondrial dehydrogenase activity of these cells using a modified MTT [3-(4,5-dimethyldiazol-2-yl)-2,5-diphenyltetrazoliumbromide] procedure according to the manufacturer’s instructions (Dojindo Molecular Technologies Inc., Rockville, MD, USA). H9c2 cells were grown in Dulbecco’s modified Eagle’s medium (DMEM) with 10% (*v*/*v*) heat inactivated fetal bovine serum, penicillin G (100 U/mL), and streptomycin (100 mg/mL) in 96-well plates at a density of 10,000 cells per well at 37 °C in a humidified 5% CO_2_ atmosphere. After 24 h of growth, we divided cells into the following groups: doxorubicin at 20 µM; nutraceutical-loaded nanoemulsions in the uncoated form: dryTom-Ne, freshTom-Ne, curc-Ne and in the chitosan-coated formulations, designated dryTom-Ne-CT, freshTom-Ne-CT, curc-Ne-CT (all at concentrations ranging from 0.5 to 5% *w*/*v* of oil) all tested in combination with 20 µM doxorubicin. We decided to use only this Doxorubicin concentration referring to several works, related to cardioprotection, in the literature [2,3]. Moreover, we tested and compared the effects of Enalapril, a common inhibitor of angiotensin-converting enzyme at 10, 25 and 50 µM as reported elsewhere [28] and Carvedilol at 1, 5 and 10 µM [29] both co-incubated with 20 µM doxorubicin. Carvedilol is a nonselective beta blocker/alpha-1 blocker used to treat mild-to-severe congestive heart failure and left ventricular dysfunction after a heart attack. In all experiments, cells were incubated for 24 h under standard conditions. Cells were then washed three times with PBS at pH 7.4 and incubated with 100 μL of an MTT solution (0.5 mg/mL in cell culture medium) for 4 h at 37 °C. Absorbance readings were acquired at a wavelength of 450 nm with the Tecan Infinite M200 plate-reader (Tecan Life Sciences Home, Männedorf, Switzerland) using I-control software. Relative cell viability (%) was calculated with the following formula [A]_test_/[A]_control_ × 100, where “[A]_test_” is the absorbance of the test sample, and “[A]_control_” is the absorbance of the control cells incubated solely in culture medium. After the evaluation of cell cytotoxicity, we measured the total protein content using the Pierce Micro BCA protein assay kit (Thermo Fisher, Milan, Italy). Briefly, the cells were washed with ice-cold PBS, and incubated for 15 min in 150 μL cell lysis buffer (0.5% *v*/*v* Triton X-100 in PBS) that included 150 μL of the Micro BCA protein assay kit reagent (prepared according to the manufacturer’s instructions). Absorbance at 562 nm was measured on a plate reader. Cytotoxicity measurements were normalized by the amount of total protein content in each well.

### 2.5. Cellular Uptake Studies

#### 2.5.1. Uptake Quantification

H9c2 cardiomyoblasts were grown in DMEM with 10% (*v*/*v*) heat inactivated FBS, penicillin G (100 U/mL), and streptomycin (100 mg/mL) at 37 °C in a humidified 5% CO_2_ atmosphere. For uptake experiments, 5 × 10^3^ cells/well were seeded in a 24-well plate and allowed to grow for 24 h. The medium was then replaced with 0.1 mL of a 0.5% oil solution of fluorescent uncoated and chitosan-coated nanoemulsions in culture medium and incubated for between 0.5 and 24 h. Cells were then washed twice with PBS (pH 7.4) and after specified time intervals, the experiments were terminated by removing the supernatant, washing the cells three times with 10 mM PBS and lysing the cells with 0.1 mL of 0.5% Triton X-100 in 0.2 N NaOH. The membrane-bound and internalized fluorescent nanoemulsions were quantified by analyzing the fluorescence of the cell lysate (λ_exc_ = 485 nm, λ_em_ = 535 nm), using a calibration curve with 0.001 up 5% oil of fluorescent nanoemulsions dispersed in a cell lysate solution (10^6^ untreated cells dissolved in 1 mL of the Triton X-100/0.2 N NaOH solution).

#### 2.5.2. Mechanistic Studies

To determine the mechanism underlying nanoemulsion internalization in H9c2 cells, we investigated the effects of following treatment: bafilomycin A1 (that inhibits endosomal acidification by inhibiting membrane ATPases, that also affects the budding of endosomal carrier vesicles from early endosomes, filipin (an inhibitor of caveolae-mediated endocytosis), nocodazol (that inhibits membrane ruffling and active [vesicular] transport by disrupting cytoplasmic microtubules), cytochalasin D (a well-known inhibitor of membrane ruffling and active [vesicular] transport that acts by disrupting actin fibers), hypertonic sucrose and potassium-free buffer (that inhibit the clathrin-mediated uptake with lower and higher selectivity, respectively), and sodium azide (that reduces active transport by inhibiting cellular respiration).In the case of fluorescent nanoemulsions, cellular uptake experiments were conducted after 4 h of incubation in the presence of these inhibitors, specifically: 0.45 M sucrose, 0.1mg/mL cytochalasin D, 1 mg /mL nocodazole, 0.1 mg/mL filipin and 2 × 10^−7^ M bafilomycin A1. In separate experiments, cancer cells were preincubated with 10^−2^ M of the metabolic inhibitor sodium azide for 30 min before the uptake of uncoated and chitosan-coated nanoemulsions and during uptake to evaluate the energy dependence of the process. With the exception of sodium azide and sucrose, which were dissolved directly in the culture medium, stock solutions of the other effectors were prepared in DMSO then diluted with culture medium to the proper concentration. H9c2cells were pre-incubated at 4 °C for 30 min with inhibitors then at 37 °C for 4 h as reported elsewhere [30]. Instead, to study the effect of intracellular potassium depletion, cardiomyocytes were rinsed twice and incubated with a potassium-free buffer solution with the following substances: 0.14 M NaCl, 0.02 M of MES buffer, 10^−3^ M of CaCl_2_ and 1 mg/mL of glucose pH 7.4 for 30 min before uptake experiments were performed in the same medium, as reported in literature [31].

### 2.6. Cellular Antioxidant Activity Following Oxygen Radical Generator Exposure

Cellular uptake of nanoemulsionsinH9c2 cells after oxygen radical generator exposure was measured as reported elsewhere [32]. In brief, H9c2cellswere incubated with 50 µL of 2,2′-Azobis(2-methylpropionamidine) dihydrochloride (AAPH) 4 µM for 10 min to simulate oxidative stress. Cells were then washed three times with PBS and then treated with nutraceutical-loaded nanoemulsions at oil concentrations ranging from 0.5 to 5% *w*/*v* of oil for 10, 20, 45, or 60 min. Gallic Acid (at 25, 50 and 100 µM), a common antioxidant, was used as positive control. Subsequently, samples were washed twice and sonicated at 10% amplitude with energy of 20 W/cm^2^ for 5 min to lyse cardiomyoblasts by sonicator (Sonics, Vibra Cell, Newtown, CT, USA). After sonication, samples were centrifuged for 30 min at 2700× *g*. The supernantant was then removed as the lysate fraction. Three different plates per compound were run for lysate fractions.

### 2.7. Detection of Intracellular Reactive Oxygen Species

The formation of intracellular reactive oxygen species was evaluated using a conventional fluorescent probe, called DCFH-DA, as described elsewhere [33]. Briefly, H9c2 cells were grown in DMEM with 10% (*v*/*v*) heat-inactivated FBS, penicillin G (100 U/mL) and streptomycin (100 mg/mL) at 37 °C in a humidified 5% CO_2_ atmosphere. Subsequently, 5 × 10^3^ cells/well were seeded in a 24-well plate and allowed to grow for 24 h. After washing twice with PBS, cells were pretreated or not with all nutraceutical-loaded nanoemulsions at oil concentrations ranging from 0.5 to 5% *w*/*v* of oil for 4 h; pretreatment also with gallic acid (at 25, 50 and 100 µM) was used as positive control. After pretreatments, cells were then incubated with 5 μM DCFH-DA in PBS for 30 min. After the DCFH-DA removal, cells were stimulated with 40 ng/mL of lipopolysaccharide (LPS) or doxorubicin at 50 nM for 12 h. Cell fluorescence was measured using a microplate spectrofluorometer (xMark Microplate, Spectrofluorometer Biorad, Milan, Italy). Intracellular antioxidant activity was expressed as percentage of control cells.

### 2.8. Lipid Peroxidation Studies

To study the protective effects of nutraceutical-loaded nanoemulsions at the membrane level of cardiomyoblasts, H9c2 cells were grown in DMEM with 10% (*v*/*v*) heat inactivated FBS, penicillin G (100 U/mL), and streptomycin (100 mg/mL) at 37 °C in a humidified 5% CO_2_ atmosphere. Subsequently, 5 × 10^3^ cells/well were seeded in a 24-well plate and allowed to grow for 24 h. Briefly, H9c2 cells were treated with doxorubicin (50 nM) or LPS (40 ng/mL) for 6 h or pretreated for 4 h with all nutraceutical-loaded nanoemulsions at oil concentrations ranging from 0.5 to 5% *w*/*v* or with gallic acid (at 25, 50 and 100 µM) as positive control. Then, cells from each group were washed three times with cold PBS, harvested with 0.25% trypsin, and centrifuged at 1000× *g* for 10 min. The supernatant was discarded and the cell pellet sonicated in cold PBS. After centrifugation (800× *g*, 5 min), the supernatant was immediately evaluated for MDA and 4-HNAcommercial kits with a spectrophotometer according to the manufacturer’s protocols (Sigma Aldrich, Milan, Italy). We measured the protein content of the cell homogenates using the Micro BCA protein assay kit (Pierce, Thermo Fisher, Milan, Italy) according to kit instructions.

### 2.9. Measurement of Nitric Oxide

To evaluate the effects of nutraceutical-loaded nanoemulsions on the release of nitric oxide from H9c2 cells we analyzed the release of nitrite, a stable product of nitric oxide in aqueous medium, using the Griess Reagent System (Promega, Madison, WI, USA) as described elsewhere [18]. Briefly, H9c2 cardiomyoblasts were grown in DMEM with 10% (*v*/*v*) heat inactivated FBS, penicillin G (100 U/mL), and streptomycin (100 mg/mL) at 37 °C in a humidified 5% CO_2_ atmosphere. Subsequently, 5 × 10^3^ cells/well were seeded in a 24-well plate and allowed to grow for 24 h. Cells were treated with doxorubicin (50 nM) or LPS (40 ng/mL) for 6h or pretreated for 4 h with all nutraceutical-loaded nanoemulsions at oil concentrations ranging from 0.5 to 5% *w*/*v*. Also, in this case, pre-incubation with gallic acid (at 25, 50 and 100 µM), a common antioxidant, was used as positive control. The culture medium was then mixed with an equal volume of sulfanilamide solution (1% *v*/*v* in 5% *v*/*v* phosphoric acid) and of N-1-naphtylethylenediamine dihydrochloride solution (0.1% *v*/*v* in water). Absorbance was measured at 540 nm with a spectrophotometer (xMark Microplate, Spectrofluorometer Biorad, Milan, Italy). Nitrite concentrations were determined from a calibration curve of standard 0.1 M sodium nitrite concentrations from 0.5 to 50 μM against absorbance.

#### 2.9.1. Intracellular Calcium Level

Doxorubicin-induced cardiotoxicity is also accompanied by an increase in intracellular calcium levels. Dysregulation of intracellular calcium concentrations is both a result and a cause of the generation of radical oxygen species. Doxorubicin induces the release of calcium from the sarcoplasmic reticulum by increasing the probability that the channel adopts the open state. An increase in intracellular calcium is not the only cause of mitochondrial calcium dysregulation, in fact, doxorubicin affects mitochondrial calcium transport thereby contributing to the increase in intracellular calcium levels. To monitor the intracellular calcium in H9c2 cardiomyoblasts, we used the fluorescence dye Fluo-3 AM, following the manufacturer’s protocol. Briefly, H9c2 cells were treated with doxorubicin (50 nM) or LPS (40 ng/mL) for 6h or pretreated for 4h with each of the nutraceutical-loaded nanoemulsions at oil concentrations ranging from 0.5 to 5% *w*/*v*. After incubation, H9c2 cardiomyoblasts were loaded with 5 µM Fluo-3 AM at 37 °C for 30 min in the dark, and then washed three times with PBS to remove excess dye. The fluorescence intensity of Fluo-3 chelated with calcium was recorded on a microplate spectrofluorometer (xMark Microplate, Spectrofluorometer Biorad, Milan, Italy) at excitation and emission wavelengths of 488 and 525 nm, respectively.

#### 2.9.2. Anti-Inflammatory Studies

The expression of IL-6, IL-8, IL-1β, IL-10, and tumor necrosis factor-alpha (TNF-α) in cardiomyoblasts was evaluated with ELISA, as described elsewhere [34]. Briefly, H9c2 cells were grown in DMEM with 10% (*v*/*v*) heat inactivated FBS, penicillin G (100 U/mL), and streptomycin (100 mg/mL) at 37 °C in a humidified 5% CO_2_ atmosphere. After incubation for 24 h and starvation in serum-free medium for 2.5 h, cardiomyoblasts were treated or not with the nutraceutical-loaded nanoemulsions at oil concentrations ranging from 0.5 to 5% *w*/*v* for 4h before exposure to LPS (40 ng/mL) for 12 h to stimulate inflammation. Subsequently, culture supernatants were collected, centrifuged to pellet any detached cells, and measured using TNF-α, IL-1β, IL-6, IL-8 and IL-10 ELISA kits according to the manufacturer’s instructions (Sigma Aldrich, Milan, Italy). The sensitivity of this method was below 10 (pg/mL), and the assay accurately detected cytokines in the range of 1–32,000 pg/mL.

#### 2.9.3. Statistical Analysis

Differences between the experimental groups were identified with a one-way analysis of variance and subsequently with Turkey’s multiple comparison test in Sigma Plot Software (Sigma, San Jose, CA, USA). *p* < 0.05 was the lowest acceptable threshold for significance.

## 3. Results

### 3.1. Synthesis and Chemical-Physical Characterization of Nutraceutical-Loaded Nanocarriers

**Figure 1:**
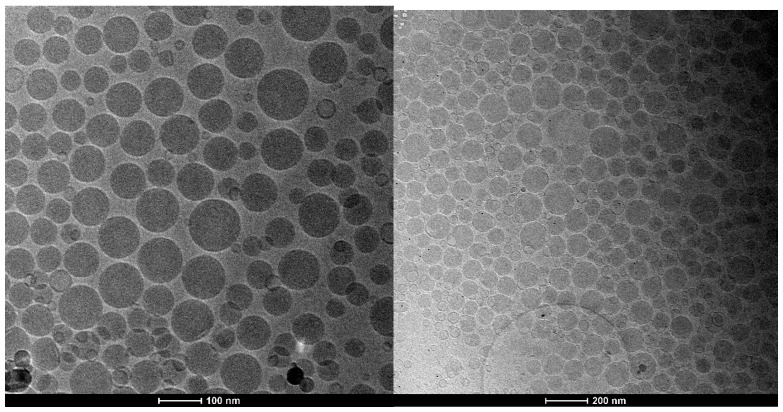
Cryo-TEM projection images of dryTom-Ne (**left**) and dryTom-Ne-CT (**right**).

Oil in water nanoemulsions in their uncoated and chitosan-coated form were produced as previously described [23]. In particular, among different possible sizes of the nanoemulsions that can be tuned with the amount of surfactant, namely egg lecithin, we chose to work with the smallest possible. Indeed, we have already demonstrated the importance of scaling down the size in terms of bioavailability once in vivo. Regarding curcumin, we reproduced the uncoated and chitosan-coated oil in water nanoemulsion that we previously used to evaluate their cardio-protective properties [16]. Then, given the well-known cardio-protective properties of lycopene, we started from tomato peel that is notoriously rich in lycopene and we applied the Naviglio solid-liquid extraction method by directly immersing tomato peel in the oil phase. After enriching the oil with the active principles contained in tomato peel, we used it to prepare the uncoated and chitosan-coated form of the oil in water nanoemulsion by applying the same procedure described above. In one extraction procedure, we used wet tomato peels directly as obtained from a tomato company (Pietro Grimaldisrl, Sant’Egidio del Monte Albino SA, Italy), and in another we lyophilized the tomato peels before extraction. In the latter case, we used an amount of peel corresponding to the starting amount of wet tomato peels. For the overall uptake study, we also used oil in water nanoemulsions again uncoated and coated with chitosan by repeating the procedure previously reported. Together with the nutraceutical encapsulating nanoemulsions we also used an FITC-loaded nanoemulsion for uptake studies. The dimensional characterization of the nanocarriers used in this study is reported in Table 2, together with the z-pot of the all systems. Thanks to the surface charge and to the narrow size distribution, the systems are stable for several months especially in the coated forms, as reported elsewhere [21,26]. As an example, the uncoated and coated nanoemulsions richest in lycopene were characterized by cryo-TEM as reported in Figure 1. It is clear from the morphological analysis the level of monodispersion of the uncoated and coated carrier and the sizes are in agreement with the DLS analysis.

### 3.2. Measurement of Lycopene Content in Tomato Peel Extracts

**Figure 2:**
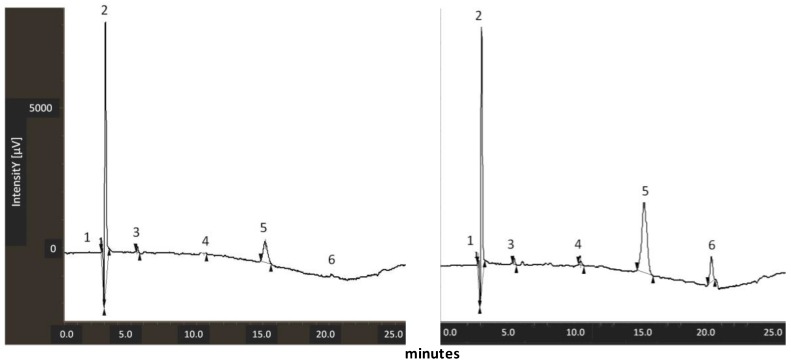
Representative chromatograms for the samples “dry tomato extract” (**left**) and “fresh tomato extract” (**right**). Lycopene is peak n. 5.

The amount of lycopene in the two samples was measured using a previously reported LC/MS method slightly modified [25]. Quantitative measurements were obtained on the basis of a calibration curve established with commercial lycopene standard samples as reported in the materials and methods section. The lycopene content was significantly lower in “fresh tomato extract” than in “dry tomato extract” (0.007 mg/mL ± 0.0005, versus 0.029 mg/mL ± 0.001). A representative chromatogram of the two samples is depicted in Figure 2. This is due to the fact that the presence of water in fresh products makes the extraction of lipophilic compounds more difficult using this method. Based on this information, for all subsequent studies, it is important to specify that the nanoemulsions are based on fresh tomato extract at 0.5%, 1% and 5% of oil contain, respectively, 0.035, 0.07 and 0.35 µg of lycopene/mL of solution. Moreover, nanoemulsions must be based on dry tomato extract at 0.5%, 1% and 5% of oil contain, respectively, 0.145, 0.29 and 1.45 µg of lycopene/mL of solution.

### 3.3. Cell Viability

**Figure 3:**
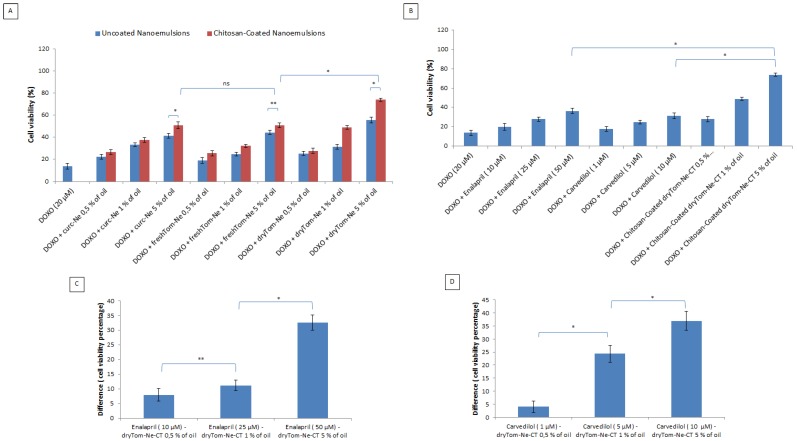
Cell viability in function of the concentration of nutraceutical-loaded uncoated and chitosan-coated nanoemulsions all tested at concentrations from 0.5 to 5% *v*/*v* of oil tested combined with doxorubicin at 20 µM. (**A**). The viability of cardiomyoblasts incubated with doxorubicin (at 20 µM) in association with Enalapril at 10, 25 and 50 µM and Carvedilol at 1, 5 and 10 µM. (**B**). Difference in the percentage of cell viability versus control cells, between (**C**) Enalapril and (**D**) Carvedilol and the best nanoemulsion (dryTom-Ne-CT) at different concentrations * *p* < 0.001; ** *p* < 0.05; ns: not significant.

As shown in Figure 3, doxorubicin treatment for 24 h decreased cardiomyoblasts viability by more than 80% but co-incubation with all the nanoemulsions used resulted in concentration-dependent cardioprotective effects. Chitosan-coated forms of nanoemulsions had the best cardioprotective properties, probably due to a better cellular uptake on cardiomyoblasts (Figure 3A). Overall cell viability was 5–20% higher in cells treated with chitosan-coated nanoemulsions than in unpretreated cells. Moreover, lycopene-rich nanoemulsions (dry-Tom-Ne) had better cardioprotective properties than did fresh-Tom-Ne-treated and curc-Ne-treated cardiomyoblasts. In fact, the viability of cardiomyoblasts treated with uncoated and chitosan-coated dryTom-Ne at 5% oil was about 45% and 60% higher, respectively versus doxorubicin-treated cells.

To determine the translational potential of these nanoemulsions in cardioprotection during doxorubicin treatment, we compared the effects of Enalapril and Carvedilol (Figure 3B–D) as common drugs used in cardio-oncology; interestingly, the best nanoemulsion formulation at the higher concentration tested seems to be 30% and 35% more effective compared to Enalapril and Carvedilol at very high concentration such as 50 and 10 µM, respectively (*p* < 0.001 for both).

### 3.4. Uptake Quantification and Mechanistic Studies

**Figure 4:**
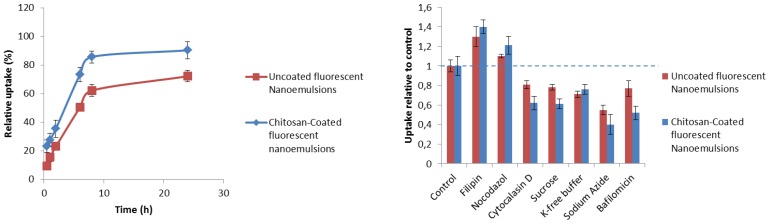
**Left**, overall cellular uptake quantification of H9c2 cells (at a density of 5 × 10^3^ cells/well) from 0.5 up to 24 h of contact with fluorescent nanocarriers at 1% *v*/*v* oil. **Right**, effect of different inhibitors on the internalization of fluorescent nanocarriers (1% *v*/*v* oil) in H9c2 cells after 4 h of incubation. The data are normalized against their controls.

As we previously reported [18], the cellular uptake of fluorescent nanocarriers is time-dependent. In the present study, after 2 h of incubation, approximately 30–35% of chitosan-coated nanoemulsions were internalized or adhered to the membrane of H9c2 cells (Figure 4). The uptake of uncoated nanoemulsions at 24 h of incubation was invariably less than 30–35% that of chitosan-coated emulsion, which corroborates the effects on cell viability described previously. After 24 h of incubation, around 90% of chitosan-coated nanoemulsions were internalized in cardiomyoblasts (Figure 4). Having established that nanocarriers are able to internalize in H9c2 cells, we complemented this study by analyzing the mechanism of cell uptake using a small library of inhibitors of general active transport processes, endosomal acidification, caveolae-mediated endocytosis, membrane ruffling and vesicular transport microtubules and actin fibers [35]. Thus, we evaluated the effects of these inhibitors on the internalization of fluorescent nanocarriers in cells. As shown in Figure 2, the nanocarriers were highly sensitive to all these factors. In detail, sodium azide inhibited endocytosis by 55–60% and cytochalasin by 35–40%. Nocodazole did not have any significant effect on H9c2, which is in line with the energy-dependent nature of the nanocarrier internalization and the involvement of stress fibers, but not of microtubules. Filipin did not significantly inhibit cellular internalization thereby excluding a caveolae-mediated endocytic mechanism of nanoemulsions uptake. Bafilomycin A1 significantly reduced endocytosis by 50%. Although hypertonic sucrose is known to inhibit also macropinocytic and caveolar uptake, the more clathrin-selective potassium-free buffer had the same inhibitory effect, indicating the clathrin-dependent endocytosis is the most likely internalization mechanism [36]. It seems that uncoated and chitosan-coated nanoemulsions have the same mechanism of endocytosis, however, chitosan-coated nanoemulsions are clearly more sensitive to the inhibitors than uncoated nanoemulsions, which indicates a clathrin-dependent endocytosis.

### 3.5. Cellular Antioxidant Activity after Oxygen Radical Generator Exposure

**Figure 5:**
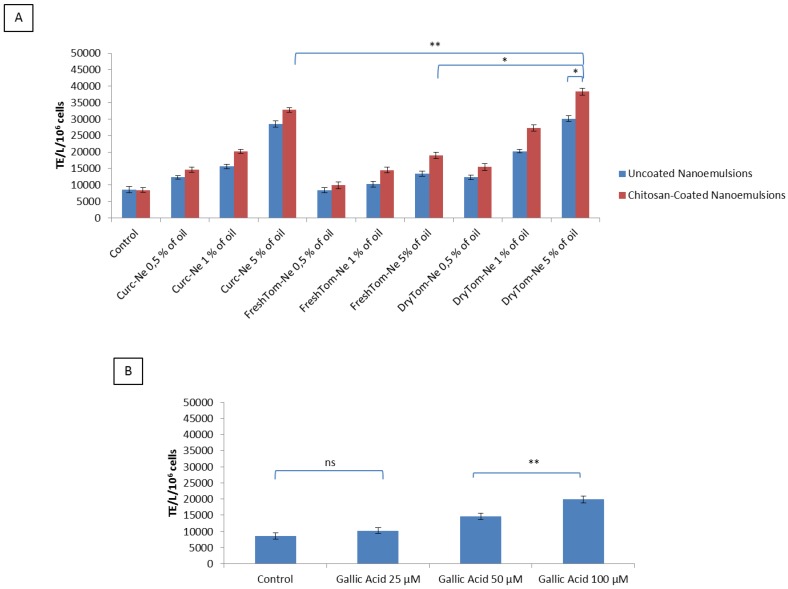
Mean antioxidant values (±SEM) of H9c2 cell lysates (TE/L/10^6^ cells). Antioxidant values of cell lysates after challenge with 50 mL of AAPH (4 mM) for 10 min and recovery with PBS (control) or chitosan-coated or uncoated nutraceutical-loaded nanoemulsions (**A**) or gallic acid (**B**) at 25, 50 and 100 µM. * *p* < 0.001; ** *p* < 0.05.

As shown in Figure 5A, the mean antioxidant capacity differed significantly between the lysates of cells treated with chitosan-coated nanocarrier sand those of uncoated nanocarriers. Specifically, treatment of H9c2 cells with dryTom-Ne exerted antioxidant activity in a concentration-dependent manner with a mean of 12,323 (±998), 20,233 (±956), 30,122 (±1023) TE/L/10 cells for 0.5%, 1% and 5% of oil, respectively. The chitosan-coated emulsion had similar behavior with even higher antioxidant activity than uncoated nanocarriers with a mean of 15,456 (±744), 27,233 (±535) and 38,344 (±898) TE/L/10 cells for 0.5%, 1% and 5% of oil, respectively. The antioxidant properties of dryTom-Ne were 10–13% higher than curc-Ne, and around 40% higher than freshTom-Ne. Gallic acid, a common antioxidant used as positive control, as reported in Figure 5B has a slight antioxidant property compared to nanoemulsions a mean of 10,245 (±886), 14,563 (±978) and 19,856 (±1023) TE/L/10 cells for 25, 50 and 100 µM, respectively.

### 3.6. Detection of Intracellular Reactive Oxygen Species

**Figure 6:**
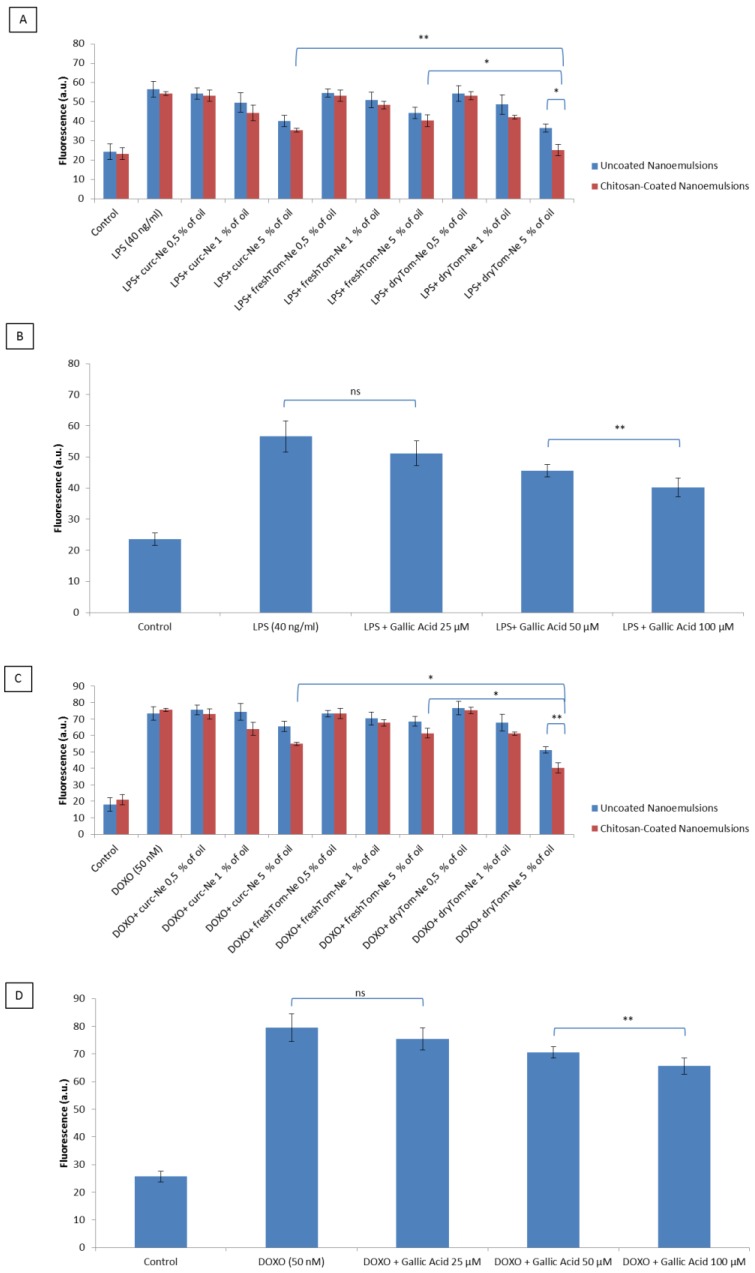
Detection of intracellular reactive oxygen species by fluorescence (a.u) in the H9c2 cell line (5000 cells/well). Cells were pretreated or not with uncoated and chitosan-coated nutraceutical-loaded nanoemulsions for 4 h before stimulation with 40 ng/mL of lipopolysaccharide (LPS) (**A**) or 50 nM of doxorubicin (**C**) for 24 h. Gallic acid was also exposed to cardiomyoblasts as positive control before stimulation with LPS (**B**) or doxorubicin (**D**). * *p* < 0.001; ** *p* < 0.05.

The H9c2 cell lysate fraction served as a model to measure the antioxidative effect of nutraceutical-loaded nanocarriers under pro-inflammatory conditions induced by lipopolysaccharides (LPS) and doxorubicin (Figure 6). As reported elsewhere [37], intracellular ROS production was higher in LPS-treated H9c2 cells due to the stimulation of the TLR4—NADPH oxidase 1 (NOX1) pathway. Treatment of cardiomyoblasts with LPS and doxorubicin increased the antioxidant properties by 50% and 55–60%, respectively, versus the control (Figure 6A). Incubation with nutraceutical-loaded nanocarriers invariably decreased oxidative status at all concentrations tested but not in a statistically significant manner at 0.5% oil, compared to untreated cells. Chitosan-coated dryTom-Ne, namely dryTom-Ne-CT, had the best antioxidant activity. Indeed, it reduced oxidative stress by 45–50%, similar to control cells, and by 45% similar to LPS- and doxorubicin-treated cells. In LPS-treated cells, the antioxidant activity of dryTom-Ne was 35% and 25% higher than that of freshTom-Ne and curc-Ne, respectively (*p* < 0.001). As shown in Figure 6B,D, Gallic acid used as positive control, had antioxidant effects both in LPS (Figure 6B) and Doxorubicin (Figure 6D) treated cells with a reduction of oxidative stress of around 26% and 21% at 100 µM, respectively, compared to untreated cells (*p* < 0.001 for both).

### 3.7. Lipid Peroxidation Studies

**Figure 7:**
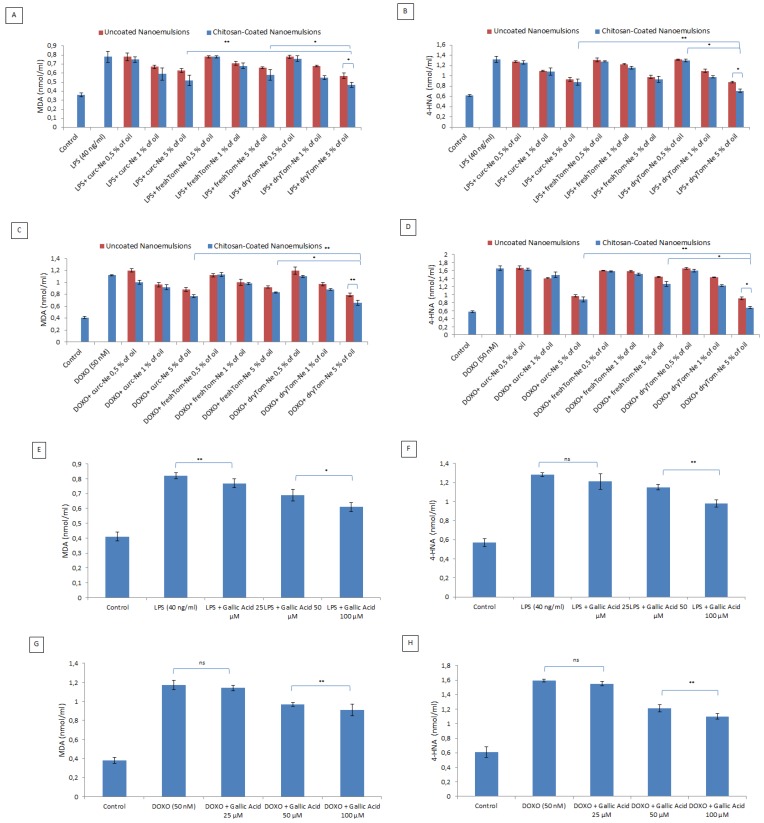
Cellular quantification of malondialdehyde (MDA) and 4HNA production under pro-inflammatory conditions and doxorubicin exposure. MDA and 4HNA (nmol/mL) production by cardiomyoblasts treated with LPS (**A**,**B**) and doxorubicin (**C**,**D**) alone or combined with uncoated or chitosan-coated nutraceutical-loaded nanocarriers at concentrations ranging from 0.5 to 5% oil. At the same condition, as positive control, we tested also the effects of gallic acid at 25, 50 and 100 µM (**E**,**F**) for LPS treatments; (**G**,**H**) for doxorubicin treatments). * *p* < 0.001; ** *p* < 0.05; ns: not significant.

As reported in Figure 7, nutraceutical-loaded nanocarriers significantly decreased the production of MDA and 4HNA by cardiomyoblasts under pro-inflammatory conditions (LPS) and chemotherapy (doxorubicin). Inhibition of lipid peroxidation is a cardioprotective strategy used to increase vascular and cardiac cell viability during anthracycline therapies. Specifically, curcumin-loaded nanocarriers, chitosan coated, at 5% oil reduced the formation of MDA and 4HNA by 31%and 23%, respectively versus LPS-treated cells (*p* < 0.001); dryTom-Nehad better lipid-antioxidant properties than the other nutraceuticals with reductions (at 5% oil) of MDA and 4HNA of 37.5% and 57%, respectively versus LPS-treated cells (*p* < 0.001). The same applies to doxorubicin-treated cells, which indicates that the same mechanism of action counteracts lipid peroxidation synthesis in the two nutraceuticals. As positive control, we analyzed effects of gallic acid as common antioxidant at the same conditions tested with nanoemulsions and, as reported in Figure 7E–H, at 100 µM, it reduced MDA and 4HNA production of around 26% and 21% in LPS treated cells and of 20% and 28%, respectively, in doxorubicin treated cells. However, based on all results obtained, chitosan-coated lycopene-enriched nutraceuticals, namely dryTom-Ne-CT, had the best biological properties of all the nutraceuticals evaluated thereby corroborating the results of the cell viability, antioxidant and cellular uptake studies.

### 3.8. Measurement of Nitric Oxide

**Figure 8:**
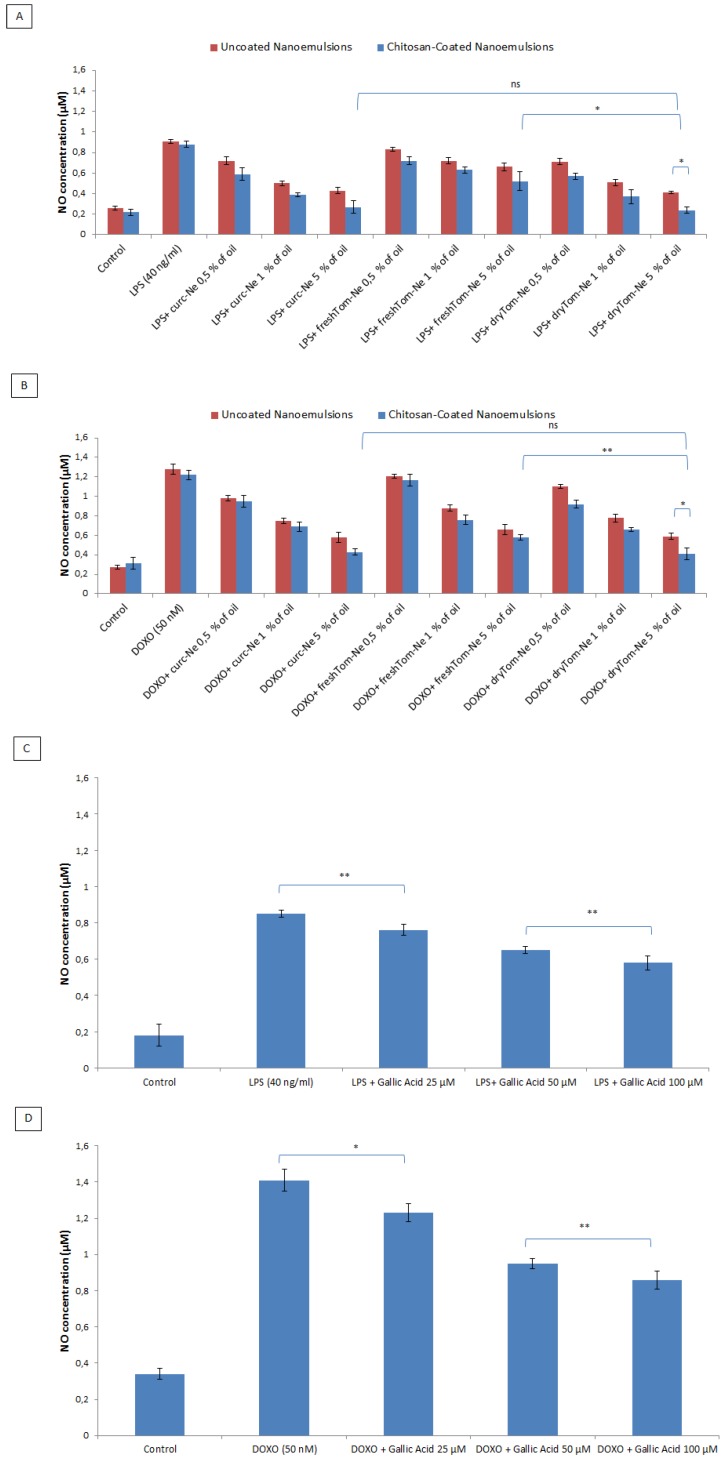
Measurement of nitric oxide (NO) production expressed as nitric oxide concentration (µM) in H9c2 cells (5000 cells/well). Cells were pretreated or not with uncoated and chitosan-coated nutraceutical-loaded nanoemulsions at 0.5%, 1% and 5% of oil 4 h before stimulation with 40 ng/mL of LPS (**A**) or 50 nM of DOXO, (**B**) for 24 h. Pretreatments were also performed by incubating cells with gallic acid, as positive control, for both LPS (**C**) and DOXO (**D**) treatments. * *p* < 0.001; ** *p* < 0.05; ns: not significant.

Nitric oxide initiates lipid peroxidation and, by interacting with superoxide anion, produces peroxynitrite which is implicated in atherosclerosis [38]. Peroxynitrites trigger lipid peroxidation, protein oxidation, nitration and activation of matrix metalloproteinases [39]. Under pro-inflammatory conditions and chemotherapy, cardiomyoblasts can produce NO. As shown in Figure 8A,B, H9c2 cells increase NO production under pro-inflammatory conditions and chemotherapy. Treatment with nutraceutical-loaded nanocarriers significantly inhibited NO production at all tested concentrations with inhibition percentages reaching 93% (very near to baseline concentrations) in the case of curc-Ne-CT and dryTom-Ne-CTat the maximum tested concentration (5% of oil). As shown in Figure 8, there was no significant difference between curcumin-loaded and high lycopene concentration nanoemulsions, for both uncoated and chitosan coated nanoemulsions, which suggests that the same mechanism of action underlies the NO inhibition of these bioactives. Also, in this experiment, the chitosan-coated nanoemulsions were more effective than the uncoated ones. As positive control, gallic acid at the maximum concentration tested (100 µM) inhibit of around 33 and 42% NO production under LPS and Doxorubicin exposure, respectively, compared to unpretreated cells.

### 3.9. Calcium Levels

**Figure 9:**
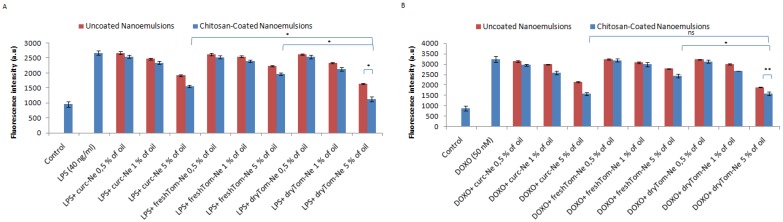
Intracellular calcium fluorescence staining in H9c2 cells (expressed as fluorescence intensity a.u). Cells were pretreated or not with uncoated and chitosan-coated nutraceutical-loaded nanoemulsions at 0.5%, 1% and 5% of oil for 4 h before stimulation with 40 ng/mL of LPS (**A**) or 50 nM of DOXO, (**B**) for 24 h. * *p* < 0.001; ** *p* < 0.05; ns: not significant.

To determine the intracellular calcium level in cardiomyoblasts during LPS or doxorubicin treatments and the biological effects of nutraceuticals-loaded nanoemulsions, we used the fluorescence probe Fluo-3 AM as reported elsewhere [40], LPS and DOXO treatment dramatically increased intracellular calcium levels in cardiomyoblasts compared to control cells (*p* < 0.001) for both. DryTom-Ne-CT treatment dose-dependently attenuated the overload of intracellular calcium in cardiomyoblastsas witnessed by the reduced fluorescence intensities of 8%, 19% and 55.6% at 0.5%, 1% and 5% (*p* < 0.001) compared to cells treated with LPS alone (Figure 9A). Similar behavior was seen for DOXO exposed cardiomyoblasts; specifically, dryTom-Ne-CT decreased calcium overload in H9c2 cells of 0.6%, 17% and 52% at 0.5%, 1% and 5% (*p* < 0.001) compared to only DOXO treated cells (Figure 9B). These results suggested that nutraceuticals-loaded nanoemulsions were able to reduce the overload of intracellular calcium in H9c2 cells induced by LPS and DOXO treatments.

#### Anti-Inflammatory Studies

**Figure 10:**
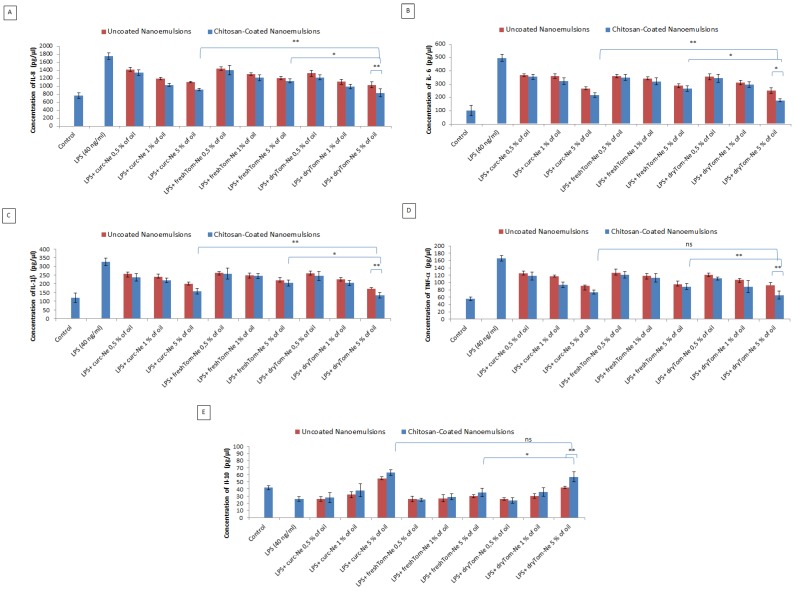
Anti-inflammatory effects of nutraceutical-loaded nanoemulsionson IL-8 (**A**), IL-6 (**B**), IL-1β (**C**), TNF-α (**D**) and IL-10 (**E**) production of cardiomyoblasts (at a density of 1.2 × 10^5^ cells/well). Cells were treated or not with 0.1 mL of a 0.5%, 1% and 5% of oil of chitosan-coated and uncoated nanoemulsions for 5 h before exposure to lipopolysaccharides (40 ng/mL) for 12 h. * *p* < 0.001, ** *p* < 0.05; ns: not significant.

Given the anti-inflammatory activity of nutraceuticals, we investigated how nutraceuticals affect IL-8, IL-6, IL-1β, IL-10 and TNF-α production in cardiomyoblasts under pro-inflammatory conditions. As shown Figure 8, LPS at a dose of 40 ng/mL significantly stimulated the production of all interleukins analyzed versus untreated cells due to their binding with Toll Like Receptor type 4 (TLR4) expressed on the membrane of cardiomyoblasts leads to up-regulation of interleukins mRNA expression and their secretion. Pretreatment with all nutraceutical-loaded nanocarriers significantly decreased the level of all molecules analyzed (Figure 10). Specifically, dryTom-Ne-CTat 5% oil decreased the levels of IL-8, IL-6, IL1β and TNFα, by 58%, 64%, 67% and 65% respectively, versus LPS-treated cardiomyoblasts (*p* < 0.001). These effects are very similar to those observed for curcumin-loaded nanoemulsions (chitosan coated) thereby indicating very strong anti-inflammatory effects. Lastly, IL-10, dryTom-Ne and curc-Ne stimulated the release of IL-10 from cells under pro-inflammatory conditions.

## 4. Discussion

Anthracyclines are powerful drugs used to treat a multitude of neoplasms; however, cardiotoxicity, which generally results from ROS overproduction, lipid peroxidation and mitochondrial damage, compromises their clinical applications. The heart and vascular microenvironments play key pathological roles in the etiology of cardiovascular disease related to some anticancer drugs and to radiotherapy. These recent observations are of extreme interest in cardioncology because several interleukins, cytokines, growth factors and hormones are crucial for cardiomyocyte survival. Specifically, IL-6 is a cytokine derived from T lymphocytes, macrophages and adipocytes, and acts via its membrane-bound or soluble receptor, stimulating C-reactive protein, fibrinogen hepatic synthesis, and joint inflammation and accelerating atherosclerosis processes. High IL-6 concentrations have been associated with an increased relative risk of myocardial infarction in healthy men. Moreover, IL-6 and its receptor levels have an early peak in the acute phase of myocardial infarction, probably due to plaque instability [41]. Moreover, recent clinical evidence supports the potential role of IL-8 in atherosclerosis, both as a marker or as a potential therapeutic target. In the field of interventional cardiology, it was suggested that increased serum levels of IL-8 after percutaneous coronary intervention could predict the development of heart failure in patients with acute myocardial infarction [42]. Another interleukin of interest in cardioncology is Interleukin-1 (IL-1) that plays an important role in the development and progression of coronary atherosclerosis and congestive heart failure. Moreover, IL-1 receptor antagonists exerted cardioprotective effects in rat myocardial ischemia-reperfusion injury and mouse viral myocarditis. Interestingly, doxorubicin treatment leads to overproduction of IL-1β in cardiac tissue, which suggests that this interleukin is involved in doxorubicin-related cardiotoxicity and apoptosis induction also by overloading calcium in cardiac cells. Moreover, IL-1 in association with nitric oxide (in the field of the NO-IL1β axis) can induce neonatal cardiac myocyte apoptosis even at very low concentrations [43].

Under pro-inflammatory conditions such as LPS exposure, fibroblasts, monocytes, macrophages and cardiomyocytes secrete TNF-α [44]. Toll-like receptor 4 (TLR4), which is the main target of LPS, is expressed throughout the body including cardiomyocytes, and it is involved in the etiology of several cardiovascular diseases; for example, cardiac dysfunctions were frequently observed in patients with sepsis and also in animals injected with LPS [45]. These findings suggest that, the heart microenvironment, seen as the inflammation-interleukin-NO-ROS axis, could play a key role in the pathogenesis of cardiotoxicity. Consequently, new cardioprotective strategies that act on this network could optimize heart protection in patients undergoing cancer treatments.

Nutraceuticals are natural bioactives that exert anti-inflammatory activities. Lycopene is a promising cardioprotective anti-inflammatory molecule that acts on the AMPK-mTOR pathway and exerts anti-atherosclerosis and anti-myocarditis effects [46]. Lycopene, a naturally occurring hydrocarbon carotenoid found in red food such as tomatoes, pink guavas and watermelons has attracted considerable clinical attention as a potential chemopreventive agent against cardiovascular disease [47]. Interestingly, lycopene regulates Mg^2+−^ATPase and maintains the Mg^2+^ balance during some anticancer therapies [48]. In addition, lycopene exerted heart-protective effects and improved cardiac function in preclinical models [49]. Curcumin suppresses the inflammatory cytokines IL-1, -1β, -6, and -8, TNF-α, and COX-2 [16]. It was recently demonstrated that curcumin decreased several markers of doxorubicin-related cardiotoxicity in preclinical models, however its biological activities are limited by its reduced bioavailability [12]. Consequently, new formulations are required to optimize bioavailability of cardioprotective nutraceuticals during doxoribincin treatment. Biodegradable nanocarriers are promising pharmacological tools to increase in vivo accumulation of drugs and/or chemo-sensitizing agents in cancer therapy both by oral or by intravenous injection as we have recently shown with similar nanocarriers [16,23].

Here we show that the nanoemulsions loaded with both curcumin and lycopene (derived from fresh or dry tomato extract) exert multiple molecular mechanisms of cardioprotection during doxorubicin treatment. The anti-inflammatory activities of the nanocarriers described herein hold potential in terms of modulating the heart microenvironment thereby providing insights for further preclinical studies, also in combination with other cardiotoxic anticancer drugs, namely anti-VEGF therapy and immunotherapies. Such studies could be valuable especially given the crucial role of interleukins in the pathogenesis of cardiac toxicity.

The results of this study demonstrate the cardioprotective effects of nanoemulsions loaded with nutraceuticals with abilities of cellular internalization with actin and ATP-dependent mechanism of uptake. However, we recognize that one limitation of our study is the use of only one type of cardiomyocyte and further investigations are required to explore the biological effects of nutraceutical-loaded nanoemulsions also in endothelial cells as well as in the co-culture of endothelial and adult cardiomyocytes. Preclinical studies are currently underway in our group to evaluate modification of left ventricular ejection fraction and factional shortening upon administration of nanocarriers during doxorubicin treatment, by using conventional murine models of cardiotoxicity often used by our research group [50].

## 5. Conclusions

Doxorubicin-induced cardiotoxicity remains a major concern for patients receiving chemotherapy. There is great interest in identifying compounds and developing drugs aimed at reducing oxidative stress and lipid peroxidation as well as inhibiting the secretion of interleukin from cardiac cells. This study shows that nutraceutical-loaded nanocarriers exert cardioprotective effects. Of particular translational interest are their effects on interleukins especially on IL1-β, considering its prognostic role in cardiology and its correlation with an increased relative risk of cardiovascular disease [48]. Nutraceuticals loaded in nanocarriers could be considered an emerging strategy with which to elicit cardioprotection from chemotherapies.

## Figures and Tables

**Table 1:** The nutraceutical-loaded and fluorescent nanoemulsions.

Nanoemulsions and Nutraceuticals Loaded	Acronym
Uncoated nanoemulsion loaded with fresh tomato extract	freshTom-Ne
Chitosan-coated nanoemulsion loaded with fresh tomato extract	freshTom Ne-CT
Uncoated nanoemulsion loaded with dry tomato extract	dryTom-Ne
Chitosan-coated nanoemulsion loaded with dry tomato extract	dryTom-Ne-CT
Uncoated nanoemulsion loaded with curcumin	curc-Ne
Chitosan-coated nanoemulsion loaded with curcumin	curc-Ne -CT
Uncoated fluorescent nanoemulsion loaded with FITC	FITC-Ne
Chitosan-coated fluorescent nanoemulsion loaded with FITC	FITC-Ne-CT

**Table 2:** Physical–chemical characteristics of nanocarriers used in this study: hydrodynamic size, polydispersity index and ζ potential.

Nanoemulsions	Mean Hydrodynamic Size (nm)	PDI	ζ-Potential (mV)
Uncoated nanoemulsion loaded with fresh tomato extract	97.09 (0.59)	0.098 (0.011)	−22.9 (1.3)
Chitosan-coated nanoemulsion loaded with fresh tomato extract	139.6 (0.85)	0.076 (0.004)	49.6 (0.5)
Uncoated nanoemulsion loaded with dry tomato extract	93.23 (0.66)	0.122 (0.020)	−23.0 (0.9)
Chitosan-coated nanoemulsion loaded with dry tomato extract	128.8 (1.01)	0.056 (0.007)	46.1 (0.5)
Uncoated nanoemulsion loaded with curcumin	105.4 (2.83)	0.118 (0.049)	−22.0 (5.6)
Chitosan-coated nanoemulsion loaded with curcumin	119.8 (0.80)	0.079 (0.010)	44.3 (1.7)
Uncoated fluorescent nanoemulsion loaded with FITC	93.83 (0.86)	0.090 (0.016)	−30.5 (1.5)
Chitosan-coated fluorescent nanoemulsion loaded with FITC	94.50 (0.75)	0.075 (0.010)	23.7 (0.2)

## References

[1] Fernandez-Chas M., Curtis M.J., Niederer S.A. (2018). Mechanism of doxorubicin cardiotoxicity evaluated by integrating multiple molecular effects into a biophysical model. Br. J. Pharmacol..

[2] Mele D., Tocchetti C.G., Pagliaro P., Madonna R., Novo G., Pepe A., Zito C., Maurea N., Spallarossa P. (2016). Pathophysiology of anthracycline cardiotoxicity. J. Cardiovasc. Med..

[3] Maurea N., Coppola C., Piscopo G., Galletta F., Riccio G., Esposito E., De Lorenzo C., De Laurentiis M., Spallarossa P., Mercuro G. (2016). Pathophysiology of cardiotoxicity from target therapy and angiogenesis inhibitors. J. Cardiovasc. Med..

[4] Simůnek T., Stérba M., Popelová O., Adamcová M., Hrdina R., Gersl V. (2009). Anthracycline-induced cardiotoxicity: Overview of studies examining the roles of oxidative stress and free cellular iron. Pharmacol. Rep..

[5] Wu K., Schwartz S.J., Platz E.A. (2003). Variations in plasma lycopene and specific isomers over time in a cohort of U.S. men. J. Nutr..

[6] Pecoraro M., Del Pizzo M., Marzocco S., Sorrentino R., Ciccarelli M., Iaccarino G., Pinto A., Popolo A. (2016). Inflammatory mediators in a short-time mouse model of doxorubicin-induced cardiotoxicity. Toxicol. Appl. Pharmacol..

[7] Hosseini A., Bakhtiari E., Mousavi S.H. (2017). Protective effect of Hibiscus Sabdariffa on doxorubicin-induced cytotoxicity in H9c2 Cardiomyoblast Cells. Iran. J. Pharm. Res..

[8] Capasso I., Esposito E., Maurea N., Montella M., Crispo A., De Laurentiis M., D’Aiuto M., Frasci G., Botti G., Grimaldi M. (2013). Combination of inositol and alphalipoic acid in metabolicsyndrome-affectedwomen: A randomized placebo-controlled trial. Trials.

[9] Maurea N., Coppola C., Ragone G., Frasci G., Bonelli A., Romano C., Iaffaioli R.V. (2010). Women survive breast cancer but fall victim to heart failure: The shadows and lights of targeted therapy. J. Cardiovasc. Med..

[10] Møller P., Loft S., Lundby C., Olsen N.V. (2001). Acute hypoxia and hypoxic exercise induce DNA strand breaks and oxidative DNA damage in humans. FASEB J..

[11] Nakamura K., Fushimi K., Kouchi H., Mihara K., Miyazaki M., Ohe T., Namba M. (1998). Inhibitory effects of antioxidants on neonatal rat cardiac myocyte hypertrophy induced by tumor necrosis factor-alpha and angiotensin II. Circulation.

[12] Shehzad A., Wahid F., Lee Y.S. (2010). Curcumin in cancer chemoprevention: Molecular targets, pharmacokinetics, bioavailability, and clinical trials. Arch. Pharm..

[13] Mascio P., Kaiser S., Sies H. (1989). Lycopene as the most efficient biological carotenoid singlet oxygen quencher. Arch. Biochem. Biophys..

[14] Karimi G., Ramezani M., Abdi A. (2005). Protective effects of lycopene and tomato extract against doxorubicin-induced cardiotoxicity. Phytother. Res..

[15] Manikandan R., Beulaja M., Thiagarajan R., Priyadarsini A., Saravanan R., Arumugam M. (2011). Ameliorative effects of curcumin against renal injuries mediated by inducible nitric oxide synthase and nuclear factor kappa B during gentamicin-induced toxicity in Wistar rats. Eur. J. Pharmacol..

[16] Vecchione R., Quagliariello V., Calabria D., Calcagno V., De Luca E., Iaffaioli R.V., Netti P.A. (2016). Curcumin bioavailability from oil in water nano-emulsions: In vitro and in vivo study on the dimensional, compositional and interactional dependence. J. Control. Release.

[17] Yucel C., Quagliariello V., Iaffaioli R.V., Ferrari G., Donsì F. (2015). Submicron complex lipid carriers for curcumin delivery to intestinal epithelial cells: Effect of different emulsifiers on bioaccessibility and cell uptake. Int. J. Pharm..

[18] Quagliariello V., Iaffaioli R.V., Armenia E., Clemente O., Barbarisi M., Nasti G., Berretta M., Ottaiano A., Barbarisi A. (2017). Hyaluronic Acid Nanohydrogel Loaded with Quercetin Alone or in Combination to a Macrolide Derivative of Rapamycin RAD001 (Everolimus) as a New Treatment for Hormone-Responsive Human Breast Cancer. J. Cell. Physiol..

[19] Albini A., Bassani B., Baci D., Dallaglio K., Gallazzi M., Corradino P., Bruno A., Noonan D.M. (2017). Nutraceuticals and “repurposed” drugs of phytochemical origin in prevention and interception of chronic degenerative disease and cancer. Curr. Med. Chem..

[20] Nam J.S., Sharma A.R., Nguyen L.T., Chakraborty C., Sharma G., Lee S.S. (2016). Application of Bioactive Quercetin in Oncotherapy: From Nutrition to Nanomedicine. Molecules.

[21] Vecchione R., Luciani G., Calcagno V., Jakhmola A., Silvestri B., Guarnieri D., Belli V., Costantini A., Netti P.A. (2016). Multilayered silica-biopolymer nanocapsules with a hydrophobic core and a hydrophilic tunable shell thickness. Nanoscale.

[22] Vecchione R., Iaccarino G., Bianchini P., Marotta R., D’autilia F., Quagliariello V., Diaspro A., Netti P.A. (2016). Ultrastable Liquid–Liquid Interface as Viable Route for Controlled Deposition of Biodegradable Polymer Nanocapsules. Small.

[23] Vecchione R., Quagliariello V., Giustetto P., Calabria D., Sathya A., Marotta R., Profeta M., Nitti S., Silvestri N., Pellegrino T. (2017). Oil/water nano-emulsion loaded with cobalt ferrite oxide nanocubes for photo-acoustic and magnetic resonance dual imaging in cancer: In vitro and preclinical studies. Nanomedicine.

[24] Naviglio D., Formato A., Scaglione G., Montesano D., Pellegrino A., Villecco F., Gallo M. (2018). Study of the Grape Cryo-Maceration Process at Different Temperatures. Foods.

[25] Naviglio D., Caruso T., Iannece P., Aragòn A., Santini A. (2008). Characterization of high purity lycopene from tomato wastes using a new pressurized extraction approach. J. Agric. Food Chem..

[26] Vecchione R., Ciotola U., Sagliano A., Bianchini P., Diaspro A., Netti P.A. (2014). Tunable stability of monodisperse secondary O/W nano-emulsions. Nanoscale.

[27] Kozukue N., Friendman M. (2003). Tomatine, chlorophyll, β-carotene and lycopene content in tomatoes during growth and maturation. J. Sci. Food Agric..

[28] Meamar R., Dehghani L., Ghasemi M., Saadatnia M., Basiri K., Faradonbeh N.A., Javanmard S.H. (2013). Enalapril protects endothelial cells against induced apoptosis in Alzheimer’s disease. J. Res. Med. Sci..

[29] Spallarossa P., Garibaldi S., Altieri P., Fabbi P., Manca V., Nasti S., Rossettin P., Ghigliotti G., Ballestrero A., Patrone F. (2004). Carvedilol prevents doxorubicin-induced free radical release and apoptosis in cardiomyocytes in vitro. J. Mol. Cell. Cardiol..

[30] Barbarisi M., Iaffaioli R.V., Armenia E., Schiavo L., De Sena G., Tafuto S., Barbarisi A., Quagliariello V. (2017). Novel nanohydrogel of hyaluronic acid loaded with quercetin alone and in combination with temozolomide as new therapeutic tool, CD44 targeted based, of glioblastoma multiforme. J. Cell. Physiol..

[31] Huang M., Ma Z., Khor E., Lim L.Y. (2002). Uptake of FITC-chitosan nanoparticles by A549 cells. Pharm. Res..

[32] Gupta-Elera G., Garrett A.R., Robison R.A., O’Neill K.L. (2012). The role of oxidative stress in prostate cancer. Eur. J. Cancer Prev..

[33] Pal A.K., Bello D., Budhlall B., Rogers E., Milton D.K. (2012). Screening for Oxidative Stress Elicited by Engineered Nanomaterials: Evaluation of Acellular DCFH Assay. Dose Response.

[34] Nagasaki T., Matsumoto H., Kanemitsu Y., Izuhara K., Tohda Y., Kita H., Horiguchi T., Kuwabara K., Tomii K., Otsuka K. (2014). Integrating longitudinal information on pulmonary function and inflammation using asthma phenotypes. J Allergy ClinImmunol..

[35] Almalik A., Day P.J., Tirelli N. (2013). HA-coated chitosan nanoparticles for CD44-mediated nucleic acid delivery. Macromol. Biosci..

[36] Fan Y., Zhang Y., Yokoyama W., Yi J. (2017). Endocytosis of Corn Oil-Caseinate Emulsions In Vitro: Impacts of Droplet Sizes. Nanomaterials.

[37] Liu X., Pei C., Yan S., Liu G., Liu G., Chen W., Cui Y., Liu Y. (2015). NADPH oxidase 1-dependent ROS is crucial for TLR4 signaling to promote tumor metastasis of non-small cell lung cancer. Tumour. Biol..

[38] Dhalla N.S., Temsah R.M., Netticadan T. (2000). Role of oxidative stress in cardiovascular diseases. J. Hypertens..

[39] Takimoto E., Kass D.A. (2007). Role of oxidative stress in cardiac hypertrophy and remodeling. Hypertension.

[40] Zhu Y., Feng Z., Cheng W., Xiao Y. (2018). MicroRNA-34a mediates atrial fibrillation through regulation of Ankyrin-B expression. Mol. Med. Rep..

[41] Bacchiega B.C., Bacchiega A.B., Usnayo M.J., Bedirian R., Singh G., Pinheiro G.D. (2017). Interleukin 6 Inhibition and Coronary Artery Disease in a High-Risk Population: A Prospective Community-Based Clinical Study. J. Am. Heart Assoc..

[42] Apostolakis S., Vogiatzi K., Amanatidou V., Spandidos D.A. (2009). Interleukin 8 and cardiovascular disease. Cardiovasc. Res..

[43] Zhu J., Zhang J., Zhang L., Du R., Xiang D., Wu M., Zhang R., Han W. (2011). Interleukin-1 signaling mediates acute doxorubicin-induced cardiotoxicity. Biomed. Pharmacother..

[44] Singh M.V., Swaminathan P.D., Luczak E.D., Kutschke W., Weiss R.M., Anderson M.E. (2012). MyD88 mediated inflammatory signaling leads to CaMKII oxidation, cardiac hypertrophy and death after myocardial infarction. J. Mol. Cell. Cardiol..

[45] Cha J., Wang Z., Ao L., Zou N., Dinarello C.A., Banerjee A., Fullerton D.A., Meng X. (2008). Cytokines link Toll-like receptor 4 signaling to cardiac dysfunction after global myocardial ischemia. Ann. Thorac. Surg..

[46] Ojha S., Goyal S., Sharma C., Arora S., Kumari S., Arya D.S. (2013). Cardioprotective effect of lycopene against isoproterenol-induced myocardial infarction in rats. Hum. Exp. Toxicol..

[47] Bansal P., Gupta S.K., Ojha S.K., Nandave M., Mittal R., Kumari S., Arya D.S. (2006). Cardioprotective effect of lycopene in the experimental model of myocardial ischemia-reperfusion injury. Mol. Cell. Biochem..

[48] Van Tassell B.W., Toldo S., Mezzaroma E., Abbate A. (2013). Targeting interleukin-1 in heart disease. Circulation.

[49] Wang X., Lv H., Gu Y., Wang X., Cao H., Tang Y., Chen H., Huang C. (2014). Protective effect of lycopene on cardiac function and myocardial fibrosis after acute myocardial infarction in rats via the modulation of p38 and MMP-9. J. Mol. Histol..

[50] Tocchetti C.G., Carpi A., Coppola C., Quintavalle C., Rea D., Campesan M., Arcari A., Piscopo G., Cipresso C., Monti M.G. (2014). Ranolazine protects from doxorubicin-induced oxidative stress and cardiac dysfunction. Eur. J. Heart Fail..

